# Deregulation of Sucrose-Controlled Translation of a bZIP-Type Transcription Factor Results in Sucrose Accumulation in Leaves

**DOI:** 10.1371/journal.pone.0033111

**Published:** 2012-03-22

**Authors:** Sunil Kumar Thalor, Thomas Berberich, Sung Shin Lee, Seung Hwan Yang, XuJun Zhu, Ryozo Imai, Yoshihiro Takahashi, Tomonobu Kusano

**Affiliations:** 1 Graduate School of Life Sciences, Tohoku University, Aoba, Sendai, Miyagi, Japan; 2 Biodiversity and Climate Research Center (BiK-F), BioCampus-Westend, Frankfurt, Germany; 3 Division of Bioscience and Bioinformatics, College of Natural Science, Myongji University Science Campus, Namdong, Cheoin-Gu, Yongin, Gyeonggi, Korea; 4 Crop Cold Research Team, National Agricultural Research Center for Hokkaido Region, Toyohira-ku, Sapporo, Japan; United States Department of Agriculture, United States of America

## Abstract

Sucrose is known to repress the translation of *Arabidopsis thaliana AtbZIP11* transcript which encodes a protein belonging to the group of S (S - stands for small) basic region-leucine zipper (bZIP)-type transcription factor. This repression is called sucrose-induced repression of translation (SIRT). It is mediated through the sucrose-controlled upstream open reading frame (SC-uORF) found in the *AtbZIP11* transcript. The SIRT is reported for 4 other genes belonging to the group of S bZIP in Arabidopsis. Tobacco *tbz17* is phylogenetically closely related to *AtbZIP11* and carries a putative SC-uORF in its 5′-leader region. Here we demonstrate that *tbz17* exhibits SIRT mediated by its SC-uORF in a manner similar to genes belonging to the S bZIP group of the Arabidopsis genus. Furthermore, constitutive transgenic expression of *tbz17* lacking its 5′-leader region containing the SC-uORF leads to production of tobacco plants with thicker leaves composed of enlarged cells with 3–4 times higher sucrose content compared to wild type plants. Our finding provides a novel strategy to generate plants with high sucrose content.

## Introduction

Growing world population in combination with climate change demand higher productivity and alternative resources for the production of food and biofuels in the future. To meet these needs agriculturally two approaches have been developed: i) extensive yield oriented selection of important traits; ii) molecular breeding for production of stress-resistant phenotypes to abiotic and biotic factors [1]. Yet another way to gain more exploitable biomass that can be used to obtain ethanol by fermentation, might be by maximizing the conversion efficiency of solar energy to soluble sugars such as sucrose [2].

Sucrose plays a crucial role not only in carbon and energy metabolism as one of the primary end products of photosynthesis but also as a signaling molecule [3], [4], [5], [6]. Smeekens and his colleagues [7] reported that sucrose negatively controls the translation of *AtbZIP11* main open reading frame (ORF) which encodes a basic leucine zipper (bZIP) type-transcription factor belonging to the group of S (S - stands for small) family in *Arabidopsis thaliana* [8]. This post-transcriptional control is termed sucrose-induced repression of translation (SIRT) [9]. The SIRT is mediated through one of the upstream ORFs (uORFs) found in the unusually long 5′-leader region of *AtbZIP11* transcript. This uORF exhibits high identity to uORFs found in 4 other genes (*AtbZIP1, -2, -44* and *-53*) of the Arabidopsis S bZIP group [10], thus it is called the sucrose controlled upstream open reading frame (SC-uORF). 

We have been studying *bZIP* homologues in various plant species and termed them as *lip19* subfamily of the bZIP gene family. The *lip19* subfamily members are phylogenetically closely related to Arabidopsis group S bZIP members [11]. They are upregulated in various abiotic stress conditions: i.e., rice *lip19* and maize *mlip15* in low temperature stress [12], [13], [14], and *Nicotiana tabacum tbz17* responds to low temperature and salt stresses [15]. Upregulation of *tbz17* in senescing leaves was also reported [16]. In these conditions, energy and hence sucrose levels decrease abruptly to limited levels, which might lead to energy deprivation condition. In Arabidopsis, it has been shown that low energy condition triggers changes in the expression of various genes via activation of KIN10/KIN11 kinases followed by the activation of group S-bZIP transcription factor genes including AtbZIP11 and AtbZIP53 [17]. AtbZIP11 and AtbZIP53 were well studied, and asparagine (Asn) synthetase gene {*ASN1,* also known as dark-inducible 6 (*DIN6*) [18]} and/or proline (Pro) dehydrogenase (*PDH*) were identified as their targets [19], [20], [21]. However, the relationship between the group S-bZIP transcription factors and endogenous sucrose has not been studied yet.

In this study we selected *N. tabacum tbz17* along with *Arabidopsis AtbZIP53* genes and, showed SIRT in *tbz17* for the first time. Additionally, SIRT in *AtbZIP53* was also confirmed by our experiments (Figure S1). Then we revealed the crucial roles of TBZ17 and AtBZIP53 to control endogenous sucrose through the generation of SIRT-insensitive *tbz17-* and *AtbZIP53-* overexpressing plants. Based on the results, we proposed a novel strategy to generate plants with enhanced endogenous sucrose levels.

## Results

### SIRT in Tobacco *tbz17*

**Figure 1:**
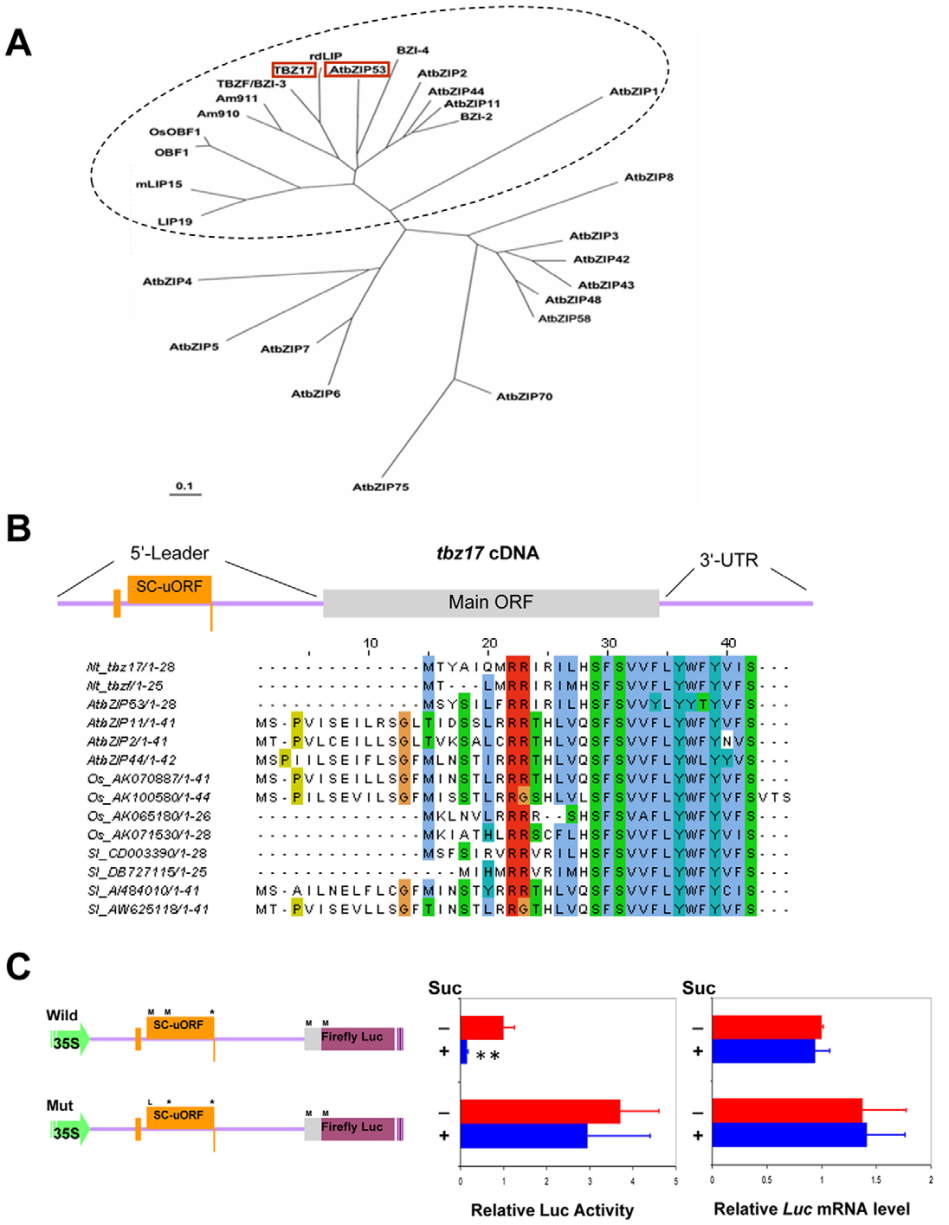
SIRT is found in tobacco *tbz17* gene. **A**, Phylogenetic relationship between 17 bZIP proteins of Arabidopsis class S [7] and LIP19 subfamily members including tobacco TBZ17. LIP19 subfamily is indicated by dotted-circle line and TBZ17 and AtbZIP53 are highlighted. The amino acid sequence alignment was constructed by the ClustalW program and the relationship was visualized by TREEVIEW program [39]. AtbZIP1 (At5g49450), AtbZIP2 (GBF5, At2g18160), AtbZIP11 (ATB2, At4g34590), AtbZIP44 (At1g75390), AtbZIP53 (At3g62420), Am910 (Y13675), Am911 (Y13676), BZI-2 (AY045570), BZI-4 (AY045572), LIP19 (X57325), mLIP15 (D26563), OBF1 (X62745), rdLIP (AB015187), TBZ17 (D63951), TBZF (identical to BZI-3, AB032478), OsOBF1 (AB185280). **B**, Structure of tobacco *tbz17* cDNA and its evolutionary conserved uORF. The first, second and third uORFs in the 5′-leader region are positioned in the second-, first- and third-frames of *tbz17* cDNA, respectively. The second uORF, here called SC-uORF, is highly conserved. The predicted amino acid sequence encoded by *tbz17* SC-uORF is aligned with those of the other group S bZIP-encoded cDNAs. *Nt*, *Nicotiana tabacum*; *At*, *Arabidopsis thaliana*; *Os*, *Oryza sativa*; *Sl*, *Solanum lycopersicum*. **C**, SIRT found in *tbz17* is mediated by its SC-uORF. The constructs used are as follows; Wild: the intact *tbz17* 5′-leader sequence (+1 to +358 of *tbz17* cDNA) was inserted between the CaMV 35S promoter and the firefly luciferase (*LUC*) gene. Mut: the start codon (ATG) of SC-uORF is mutated to Leu codon (TTG) and the second Met codon (ATG) is replaced by stop codon (TAA) by site-directed mutagenesis. The constructs were delivered into *Arabidopsis thaliana* rosette leaves (collected from 3 week-old-seedlings). The bombarded leaves were incubated in half-strength MS media for 20 h with or without 6% sucrose. The relative *LUC* mRNA levels and LUC activities were analyzed. The error bar represents the SD. Asterisk indicates significant differences that were observed due to sucrose treatment (Student’s t-test: **P<0.01).

**Figure 2:**
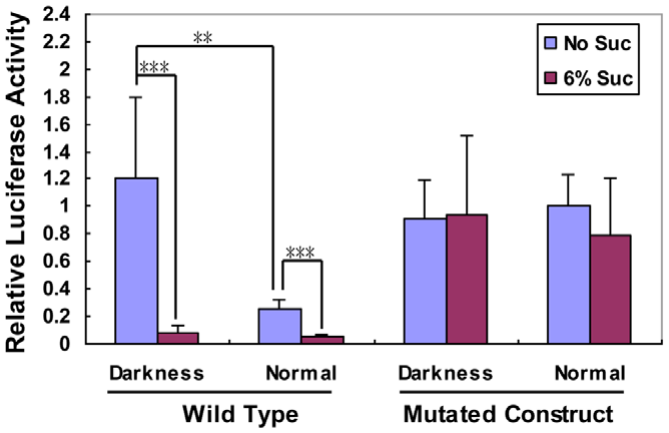
Wild type SC-uORF supresses the activity of Luc reporter in leaves kept in normal light condition but not in the dark condition. The mutated SC-uORF lost such regulation ability. Leaves of intact Arabidopsis plants that were exposed to normal light regime or kept in darkness for 24 h, respectively, were detached and bombarded with the corresponding SC-uORF luciferase constructs, WT construct and mutated one (shown in Fig. 1C). The detached leaves were incubated in half-strength MS solution with or without containing 6% sucrose for 20 h in darkness and then the Luc activity was assayed. The error bar indicates the SD. Asterisk indicates significant difference (Student’s t-test: **P<0.01; ***P<0.001).

The *lip19* subfamily members which include tobacco *tbz17*, are phylogenetically closely related to the *Arabidopsis* group S of bZIP genes carrying SC-uORFs (Figure 1A) [11]. *tbz17* cDNA contains 3 uORFs in its 5′-leader of which the second uORF shows high identity to the SC-uORF [15] (Figure 1B, Figure S2). To address the question of whether *tbz17* has a SIRT mechanism, we firstly performed a transient assay using *Arabidopsis* mature rosette leaves and a luciferase (*LUC*) reporter gene construct. In the wild type (Wild) construct, the LUC activity was profoundly inhibited by the presence of 6% sucrose (Figure 1C), whereas, in the mutated construct (Mut), in which the start codon and the second Met codon of the SC-uORF were changed to Leu codon (TTG) and stop codon (TAA), respectively, the LUC activity was not repressed by sucrose (Figure 1C). The result indicated that SIRT mediated by the SC-uORF exists in *tbz17.* Next we assayed the relative LUC activity in *Arabidopsis* rosette leaves that have been exposed to different light conditions; one was 4 h-light in a 16 h light/8 h dark photocycle (normal condition) and the other was complete darkness for 44 h (dark condition, see Materials and Methods). In dark condition, the LUC activity with Wild construct was almost comparable to the one with Mut, while, in normal light condition, the LUC activity with Wild construct was ca. 75% lower compared to that with Mut construct (Figure 2). It suggests that the translation of *tbz17* main ORF is controlled by endogenous sucrose levels. SIRT in *tbz17* was further confirmed by a transgenic approach. We generated transgenic tobacco plants which carried the CaMV 35S promoter-driven *tbz17* intact 5′-leader sequence translationally-fused to a *GUS* reporter gene. Histochemical GUS staining in two independent transgenic lines (#1–1 and #3–1) was specifically repressed by sucrose in a dose-dependent manner but not by glucose or by fructose (Figure S3B). In those seedlings, the *GUS* transcripts were detected at the similar levels whatever sugars were present, while the GUS protein levels were decreased in the presence of sucrose (Figure S3C, D). The evidence indicates that SIRT functions in tobacco *tbz17* through the SC-uORF, suggesting that SIRT is a common phenomenon in plants.

### Generation of *N. tabacum* plants overexpressing SIRT-insensitive *tbz17*

**Figure 3:**
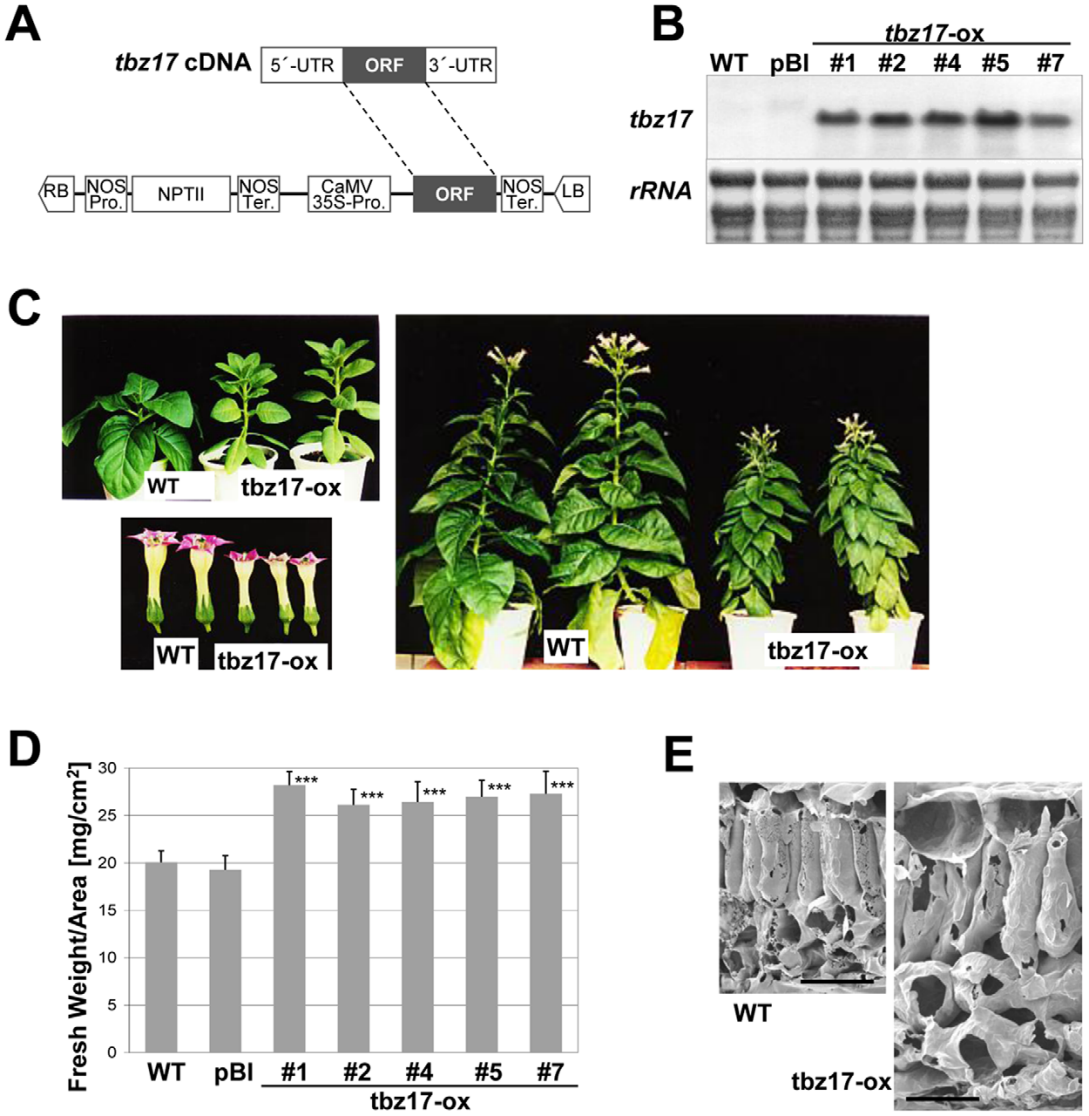
Generation of SIRT-insensitive *tbz17*-ox tobacco plants. **A**, Structure of the binary vector construct lacking the 5′-leader region required for translational repression by sucrose. *tbz17* main ORF is inserted into pBI121 vector (see Materials and Methods). **B**, Expression of *tbz17* in five independent transgenic tobacco lines. Total leaf RNA was separated on agarose gels, transferred onto nylon membrane and hybridized with the ^32^P-labelled *tbz17*-ORF cDNA-fragment. *rRNA* stained by methylene blue solution is shown as a loading control. WT, non transgenic tobacco plants; pBI, control transgenic tobacco plants; *tbz17*-ox, transgenic tobacco plants transformed by the construct shown in **A**. **C**, Representative growth phenotypes of tobacco plants overexpressing *tbz17*. Left upper image, young seedlings; left bottom image, flowers; right image, mature stage of tobacco plants. **D**, *tbz17*-ox plants have thicker leaves in relative to WT and the control transgenic plants. Discs from leaves of similar growth stage of WT, pBI and *tbz17*-ox plants were punched out with a cork borer and their fresh weights were measured. The error bar represents the SD. The differences between WT/pBI and *tbz17*-ox lines were highly significant as calculated by Students *t*-test (***P<0.001). **E**, Vertically dissected leaf images of WT and *tbz17*-ox plants. Leaf sections were observed by cryo-SEM. Bar = 50 µm.

In general, the primary role of highly conserved uORFs seems to be translational regulation of the downstream main ORFs mediated through specific metabolites in a feedback manner: arginine [22], polyamine [23], [24], [25] and choline [26]. These facts inspired us to examine whether TBZ17 is involved in sucrose metabolism. For that purpose, we generated transgenic tobacco plants constitutively expressing the *tbz17* ORF lacking the 5′-leader containing the SC-uORF under the control of the CaMV 35S promoter (Figure 3A). Translation of the transgene-derived *tbz17* may be SIRT-insensitive in these transgenic plants which are referred as *tbz17*-ox lines. The constitutive expression of transgene-derived *tbz17* was confirmed in 5 independent lines by RNA blot hybridization (Figure 3B). The *tbz17*-ox plants showed a clear phenotype of smaller and pale-green leaves in younger stage and their vegetative growth was slightly slower compared to wild type (WT)- and the control transgenic (pBI) plants. The sizes of flowers of the *tbz17*-ox plants were also smaller compared to WT plants (Figure 3C); however, they set fertile seeds. Another prominent feature of the *tbz17*-ox plants was increased fresh weight per leaf area (cm^2^) (Figure 3D), suggesting that *tbz17*-ox plants have thicker leaves compared to WT and pBI. This postulation was based on the evidence from cryo-scanning electron microscopy method (Figure 3E). Leaves of *tbz17-*ox plants were about 1.5-fold thicker than WT and pBI. Both mesophyll- and parenchyma-cells were enlarged in *tbz17-*ox plant leaves.

### Sucrose accumulation in *tbz17-*ox plants

**Figure 4:**
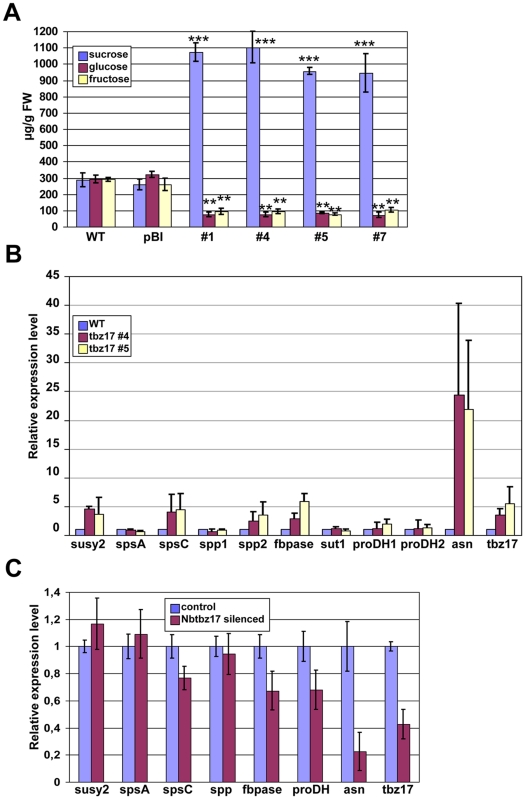
Sucrose accumulation in *tbz17*-ox plants and correlation analysis between *tbz17* and sucrose synthesis genes. **A**, Sugar contents in WT, control transgenic (pBI) and *tbz17*-ox tobacco plants. The differences in sucrose, glucose and fructose contents between WT/pBI and tbz17-ox lines were highly significant as calculated by Students t-test **B**, Quanitative real-time RT-PCR analysis on sugar-metabolizing genes in *tbz17*-ox tobacco plants. **C**, Quanitative real-time RT-PCR analysis on sugar-metabolizing genes in *Nbtbz17*-silencing *Nicotiana benthamiana*. The error bar represents the SD. *P<0.05; **P<0.01; ***P<0.001.

We hypothesized that one possible reason to have enlarged cells was the constitutive accumulation of some compatible solutes and, in this particular case, accumulation of sucrose. Thus, we measured the contents of sucrose, glucose and fructose, in 4 independent *tbz17*-ox plants and those of WT and pBI. All the *tbz17*-ox lines contained about 3- to 4-fold higher sucrose content relative to WT and pBI while glucose and fructose contents were reduced in *tbz17*-ox plants compared to WT and pBI (Figure 4A). Sucrose biosynthesis in higher plants is catalyzed by the sequential reaction of sucrose phosphate synthase (SPS) and sucrose-6′-phosphate phosphatase (SPP). The activity of SPS, a key enzyme of the pathway, is regulated at multiple levels; i.e., feedback regulation by positive or negative allosteric effectors and by phosphorylation [3], [27], [28]. In spite of the fact that sucrose synthesis is highly regulated at the post-translational level, we investigated the expression of genes which are involved in sucrose-metabolism considering that *tbz17* encodes a transcription factor. As mentioned earlier, target genes of AtbZIP11 have been identified as *ASN1* and *PDH2* [19]. One of the target genes of AtbZIP53 is *PDH2* [20]. In this context, we included *N. tabacum ASN* gene (accession number AY061820) and two *PDH* genes (accession numbers AY639145 & AY639145) in the qRT-PCR analysis of *tbz17*-ox and wild type plants. As seen in Figure 4B, *ASN* gene was highly upregulated in the *tbz17*-ox plants, whereas the expression of two *PDH* genes was not much changed compared to WT. We could tentatively conclude that one of the TBZ17-target genes is, in fact, *ASN*. It should be noted that *tbz17* expression is senescence-associated [16] and the expression of *DIN6*, identified as *ASN*, is darkness-induced and sucrose-repressed [18], [29]. Interestingly, transcripts for fructose 1, 6-bisphosphatase (*FBPase*) gene, class C sucrose phosphate synthase gene (*SPSC*) [30], sucrose phosphate phosphatase 2 gene (*SPP2*) and sucrose synthase 2 gene (*SuSy2*) accumulated 4- to 6-fold in both lines, #4 and #5, of the *tbz17*-ox plants compared to those of WT (Figure 4B). To further confirm the correlation between the levels of *tbz17* expression and *ASN*, *PDH* and sucrose-synthesizing genes, a virus-induced gene-silencing (VIGS) approach was taken. *N. benthamiana* plants in which *Nbtbz17* (*tbz17* ortholog of *N. benthamiana*) was silenced using VIGS method showed about 80% reduced expression of the *ASN* gene, and ca. 20–30% reduced expression of *PDH*, *FBPase* and *SPSC* genes, respectively (Figure 4C). The results indicated that the transcripts’ levels of *ASN* and sucrose synthesizing genes, especially *FBPase* and *SPSC* genes, positively and tightly correlated with the levels of *tbz17* transcripts. In parallel, we generated transgenic Arabidopsis plants overexpressing *AtbZIP53* ORF lacking its 5′-leader region (Methods S1). The transgenic Arabidopsis plants generated with this construct are referred as *AtbZIP53-*ox. Independent lines (#10, #12 and #22) were further analyzed (Figure S4A-C). Sucrose contents in *AtbZIP53*-ox plants are 1.5- to 2.5- fold higher compared to WT plants and, in contrast, glucose and fructose contents decreased in *AtbZIP53*-ox (Figure S4D). In relation to this, two *SPS* genes, class A of *SPS1* (*At5g20280*) and class C of *SPS4* (*At4g10120*), were upregulated in all *AtbZIP53*-ox plants (Figures S3E and S4). It should be noted that Arabidopsis SPS4 belongs to the same clade to which tobacco SPSC belongs (Figure S5). Chen et al. [30] reported that *N. tabacum SPSA* and *SPSB* are expressed in whole plant body and in reproductive organs (anther and ovary), respectively, while *SPSC* is specifically expressed in source leaves under physiological condition. Therefore, the upregulation of this class C *SPS* gene may contribute to higher sucrose content. Next question was whether TBZ17 transactivated *SPSC* gene directly or indirectly. To address this issue, we took an indirect approach and tested whether AtbZIP53 directly transactivates 4 kinds of *SPS* genes. The results showed that AtbZIP53 transactivates the *ASN* gene but not all *SPS* genes (Figure S6), suggesting that upregulation of tobacco *SPSC* in *tbz17*-ox plants is a secondary effect.

## Discussion

We have shown that a tobacco bZIP gene, *tbz17*, retains SIRT mechanism mediated by a conserved SC-uORF (Figure 1). This is a first report on SIRT beyond *Arabidopsis* genus, suggesting that SIRT is a common phenomenon in higher plants. Furthermore, the translation of *tbz17* main ORF was decreased to ca. one-forth in the leaves exposed to normal light condition compared to the one of the leaves placed under complete darkness (Figure 2). This difference was not observed with the SC-uORF-mutated construct (Figure 2). Thus, the reasons for the difference in translation might be due to elevated endogenous sucrose levels causing SIRT, suggesting that sucrose is a physiological effector to control the translation of *tbz17* main ORF.

Hanfrey et al. [25] revealed that the sequence conserved uORFs control the translation of the main ORF encoding *S*-adenosylmethionine decarboxylase (SAMDC) in response to cellular polyamine contents in Arabidopsis. In this post-transcriptional regulation system, the uORFs are polyamine-sensors and the system contributes to polyamine homeostasis in the cells. Similarly to this feedback mechanism, it is likely that the SC-uORF functions as a sucrose-sensor and that SIRT contributes to sucrose homeostasis. To assess this hypothesis, we introduced the SIRT-insensitive *tbz17* construct, deleting its 5′-leader region containing SC-uORF, into tobacco plants. The resulting tobacco plants had thicker leaves composed of enlarged cells (Figure 3). In de-regulated condition of SIRT (at least for the introduced ‘*tbz17*’ gene), the transgenic tobacco plants contained 3- to 4-fold higher sucrose in the cells (Figure 4A). Taking a similar strategy for *AtbZIP53*, the transgenic Arabidopsis contained 1.5- to 2.5-fold higher sucrose (Figure S4). These combined results support our hypothesis.

**Figure 5:**
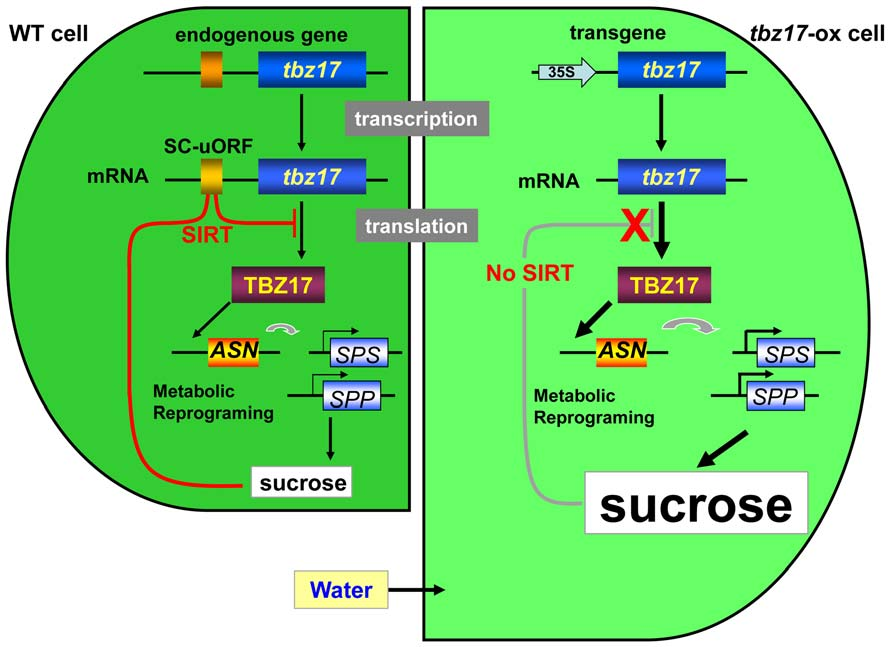
A proposed model to explain how *tbz17*-ox plant cell accumulates higher sucrose and becomes enlarged. In WT tobacco cells, when the endogeneous *tbz17* gene is transcribed and its transcript contained SC-uORF. The gene product, TBZ17, transactivates *NtASN* gene and then indirectly activates *SPS* and *SPP* genes through metabolic reprogramming, which turns on the sucrose accumulation. If the SC-uORF of *tbz17* transcript senses higher sucrose concentration, SIRT is induced, therefore, cellular sucrose concentration is maintained in a certain range. *tbz17*-ox plant cells (1.5-fold enlarged cell) contain not only the endogenous *tbz17* gene but also the transgene `*tbz17*’ which transcript does not contain SC-uORF. In the latter cells, the gene product TBZ17 directs the transactivation of *ASN* gene and indirectly upregulates `*SPS* and *SPP*’ genes. Increased sucrose concentration represses the translation of endogenous transcript carrying SC-uORF but not the transgene-derived transcript, thus sucrose concentration increased more compared to WT cells. To adjust the high osmotic pressure due to high sucrose concentration, the cell adsorbs more water and become enlarged.

Arabidopsis SnRK1 (SNF1-related protein kinase 1) -like kinases, KIN10 and KIN11, function as central signal integrators for adapting to low energy condition such as darkness, low sugar and stress conditions [17]. The KIN10 kinase-signal pathway was mediated by a specific subset of group S bZIPs including bZIP53, and activate *DIN6* ( = *ASN1*) transcription. *ASN1* transcriptional activation was blocked by sugars [18]; i.e., sucrose and glucose because those sugars inhibited KIN10/KIN11 activation [17]. To superimpose the KIN10 kinase signal pathway to tobacco plant, the order of signaling is predicted to be: KIN10-like SnRK1 kinase(s) – TBZ17 – *ASN* and/or *PDH*. In fact, the transcription of *ASN* but not *PDH* was positively correlated with the levels of *tbz17* transcripts in *tbz17*-ox plants and in *Nbtbz17*-silenced *N. benthamiana* plants (Figures 4B, C). In addition, the transcription of *FBPase* and class C *SPS* genes was correlated with the levels of *tbz17* transcripts. Even in *AtbZIP53*-ox plants, classes A and C *SPS* genes were upregulated (Figures S4, S5). We predict that the enhanced transcription of the class C member of *SPS* gene family contributes to sucrose accumulation. The transcription assay shows that AtbZIP53 transactivates *ASN* but not all of *SPS* genes (Figure S6), suggesting that the transcript accumulation of classes A and C *SPS* genes in *AtbZIP53*-ox plants occurred in an indirect manner. Analysis of the global gene expression regulated by KIN10 showed that it controls divergent metabolic reprogramming in promoting catabolic processes and suppressing anabolic processes [17]. Recently, Dietrich et al. [31] showed that the heterodimer composed of AtbZIP1 and AtbZIP53 directly binds to the G-box-like sequence of the promoters of *ASN1* and *PDH* genes and causes the changes in Pro, Asn and branched-chain amino acid metabolism to adapt to low energy stress. Taking into account all the information, we propose a model for explaining sucrose accumulation in *tbz17*-ox plants (Figure 5); in wild tobacco plant cells, TBZ17 transactivates *ASN* and induces metabolic reprogramming, which turns into activation of the ‘sucrose synthesis pathway’, and if the end product sucrose reaches a upper threshold, SIRT is operated and suppresses the translation of TBZ17 in a feedback regulation. This sucrose-sensing circuit regulates a certain range of sucrose content in the cells. In contrast, in SIRT-insensitive *tbz17*-ox plants, even if sucrose contents reach an upper level, SIRT does not operate and thus the TBZ17 translation continues. Notable modification of SPS is reversible phosphorylation that inactivates the enzyme [27], [28]. In spinach, serine-158 of SPS is phosphorylated by SnRK1 kinase(s). It was shown that KIN10 is able to phosphorylate SPS at serine-158 *in vitro*, while KIN10 was inhibited by high concentrations of sucrose [17]. So once cellular sucrose reaches higher levels, KIN10-like SnRK1 kinase(s) may be inactivated and SPS may stay active because the dephosphorylated form is major. A similar trend of sucrose accumulation was observed in *AtbZIP53*-ox plants, but the level of sucrose accumulation was smaller compared to that of *tbz17*-ox plants. The upper threshold for sucrose content in Arabidopsis may be lower compared to that of tobacco [32]. We could argue that the introduction of SIRT-insensitive group S bZIP genes leads to sucrose accumulation in the host plant cells.

In relation with this, it is worthy to note that transcriptomic analysis using sugarcane (*Saccharum officinarum*) revealed that the expression of genes encoding sucrose transporter, ornithine aminotransferase (OAT) and ASN is positively correlated with sucrose content [33]. OAT and ASN are involved in Pro and Asn metabolism, respectively. *Dehydrin*, *LEA* and *PDH* transcript levels were also higher in the higher sucrose-contained sugarcane culms. Iskander et al. also used several genotypes of sugarcanes differing in sucrose contents and drew a similar conclusion [33]. While the adaptation mechanism to high sucrose content in sugarcane is still unclear, it should be emphasized that most of the related genes were also upregulated in high sucrose-contained tobacco plants.

To conclude, our finding provides a novel strategy to generate high-content sucrose plants. Genes coding for the type of hypothetical sucrose-sensitive bZIP transcription factors investigated in this work are present in at least 3–4 copies per plant (Figure S2). Theoretically, our proposed strategy might be applicable to the genes belonging to the group of S *bZIP* genes. Specifically, the similar engineering of rice, *lip19,* is of interest. Plants transformed with the engineered *bZIP* gene might be useful as a novel source of biofuel production. Finally, the same strategy of transgenic expression of the *bZIP* homologue lacking the regulatory 5′-leader but under control of a fruit-specific promoter might result in a production of fruits with enhanced sucrose content. Such an approach is currently under way with tomato plants.

## Materials and Methods

### Plant materials and growth condition

*N. tabacum* cv. Xanthi nc and *N. benthamiana* were grown in soil at 25°C or at 23°C under a 16 h light/8 h dark photocycle, respectively. *A. thaliana* (ecotype Columbia Col-0) was grown in soil at 22°C under a 16 h light/8 h dark photocycle.

### Transient ‘SIRT’ assay

The basal vectors pUC19K and pUC19K-Luc were constructed as follows: Cauliflower mosaic virus (CaMV) 35S promoter- and Nos terminator- fragments derived from pBI221 (Clontech) were subcloned into *Hin*dIII-*Sma*I sites and *Sac*I-*Eco*RI sites of pUC19, respectively, resulting in pUC19K. Firefly luciferase (*LUC*) gene sandwiched with *Sma*I and *Sac*I sites was inserted into the respective sites of pUC19K, yielding pUC19K-Luc. Then, intact 5′- leader sequence of *tbz17* cDNA [15] in size of 361 bp was amplified by using a primer pair (forward, 5′-AGCTCTAGATGATCTTTTTTGTTAATACCT-3′ and reverse, 5′-TGACCCGGGAGCCATGTCGATTGATA-3′, the underlined *Xba*I and *Sma*I sequences were incorporated for cloning purpose, respectively). The sequence-verified fragment was digested with *Xba*I and *Sma*I, and inserted into the corresponding sites of pUC19K-Luc. The resulting plasmid was used as ‘wild type’ reporter construct of 5′-leader of *tbz17* (see Figure 1C). The mutant construct was made by inserting point mutations through PCR in which the start codon (ATG) of the 2^nd^ uORF of *tbz17* 5′-leader sequence was changed to a Leu codon (TTG) and the 2^nd^ methionine codon of the 2^nd^ uORF was changed to a stop codon (TAG). The two reporter constructs were used for transient assay experiments. Gold particles were coated with the reporter plasmid and the reference plasmid pPTRL [34], in which Renilla luciferase (*RLUC*) gene is placed under the control of the CaMV 35S promoter. Mature rosette leaves of *A. thaliana* were bombarded with the coated gold particles using a PSD-1000/He Particle Delivery System (Bio-Rad). Transformed leaves were kept on half strength Murashige-Skoog (MS) media supplemented with or without 6% sucrose for 20 h under darkness to avoid fluorescence quenching. Darkness treatment of Arabidopsis leaves was applied as follows: leaves were kept in darkness 24 h prior to bombardment and 20 h after bombardment, then LUC activity was measured. To normalize the efficiency of particle bombardment, relative luciferase (LUC/RLUC) activities were measured according to the protocol of the Dual-Luciferase Reporter Assay System (Promega) by using a luminescence reader (Lumat LB9507, Berthold Japan, Tokyo). The experiment was repeated three times with three replicates per time.

### Generation of transgenic tobacco plants overexpressing *tbz17*

A fragment of the binary vector pBI121 (Clontech), encompassing GUS coding region and Nos terminator, was eliminated by restriction with *Bam*HI and *Eco*RI and replaced with the *Bam*HI-*Eco*RI Nos terminator fragment derived from pCaMV-neo (provided by Dr. Virgina Walbot), yielding pBI001. The coding region of *tbz17* cDNA [15] was amplified by PCR with a primer pair (*tbz17*-F, 5′- GCGGATCCATGGCTTCCACTCAGCAAGC-3′ and *tbz17*-R, 5′-CGGGATCCTCAAAACAGCAACATATCAGAAG-3′), where *Bam*HI sites are shown as underlined. The *Bam*HI-digested fragment was inserted into pBI001, and the recombinant with sense orientation of *tbz17*-main ORF was named pBI-tbz17. *tbz17*-overexpressing tobacco plants were generated by infection with *Agrobacterium tumefaciens* strain LBA4404 [35] carrying pBI-tbz17.

### Northern blot analysis

Total RNAs were isolated according to the method of Nagy et al. [36]. Aliquots (20 µg each) were separated by electrophoresis on formaldehyde-1.0% (w/v) agarose gels and blotted onto Hybond N^+^ membranes (GE Healthcare) in 20×SSC. The ^32^P labelled-fragment covering the main ORF of *tbz17* cDNA or *AtbZIP53* cDNA, respectively, was used as a probe. Hybridization was performed as described previously [37].

### Cryo-scanning electron microscopy

For scanning-electron microscopy (SEM) leaves of tobacco plants were frozen in liquid nitrogen and subsequently freeze-dried. Sections of ca. 2 mm edge length were cut, placed on a carbon grid and sputtered for 5 min with gold. Examinations were performed with a Hitachi S-4500 scanning electron microscope.

### Determination of sugar contents in plants

The contents of sucrose, glucose and fructose were determined by enzyme-coupled reactions, based on the measurement of NADPH absorption at 340 nm, using the Sucrose/D-Glucose/D-Fructose kit (r-biopharm, Darmstadt, Germany), as described by the manufacturer. Fresh leaf material was ground with a mortar and a pestle under liquid nitrogen to fine powder and 200 mg of the powder was weighed into a microcentrifuge tube, briefly homogenized with 600 µl of distilled water and immediately boiled for 10 min in a water bath. After centrifugation (20,000 × *g*, 10 min at 4°C), 100 µl of the supernatant was used in the assay with a spectrophotometer (Hitachi U-2900).

### Quantitative real-time reverse transcription-polymerase chain reaction (qRT-PCR) analysis

Quantitative real-time RT-PCR was performed with FastStart Universal SYBR Green Master (ROX) (Roche Applied Science, Mannheim, Germany). First-strand cDNA was synthesized with Rever Tra Ace (Toyobo Co. Ltd., Osaka, Japan) and oligo-dT primers. The subsequent quantitative PCR was performed in a StepOne real-time PCR system (Life Technologies Japan, Tokyo, Japan) using the appropriate primer pairs (see Table S1). The steady state levels of the transcripts were determined in relative to the levels of the transcripts of a housekeeping gene encoding ribosomal protein L-25 as an internal control. All quantitative RT-PCR experiments were performed with biologically independent samples at least three times.

### Virus-induced gene silencing (VIGS) in *N. benthamiana*

For VIGS, the method described was employed [38]. The fragment of *Nbtbz17* cDNA (*tbz17* ortholog of *N. benthamiana*) was amplified with the primer pair (forward, 5′-ATGAGCTCCGTATTCTGCACTCTTTCTCAGTA-3′ and reverse, 5′-TTTCTAGAGTTCAATGAATTCAAACGTTCAGT-3′, *Sac*I and *Xba*I sites were underlined, respectively). The resulting 500-bp-fragment was cloned into the pTRV2 vector. *Agrobacterium* cultures containing pTRV1, and pTRV2 or its derivative plasmid were similarly mixed in a 1∶1 ratio, and were infiltrated into the lower leaf of 4-leaf stage plants using a needle-less syringe.

### Statistical analysis

The data analysis was performed using the statistical tools (Student’s *t* test) of Microsoft Excel software.

## Supporting Information

Figure S1 **Confirmation of SIRT in Arabidopsis** ***AtbZIP53*** **cDNA.** **A**, Schematic drawing of the cloned fragment in binary vector construct for transformation. *AtbZIP53* genome DNA fragment, spanning from -919 to +552, was inserted into *Pst*I and *Bam*HI-digested pBI101 vector (Clontech), yielding pAtZIP53G. The recombinant was a GUS-translational fusion construct. **B**, Histochemical staining of the *AtbZIP53* promoter-GUS transgenic Arabidopsis seedlings. Among the transgenics, two independent lines were used for assay. 5-day-old Arabidopsis seedlings were incubated with or without 20 mM or 100 mM sucrose for 2 days, then rinsed with distilled water twice, and subjected to histochemical staining according to the procedure described by Jefferson (1987). **C** and **D**, *GUS* transcript levels (**C**) and GUS protein levels (**D**) in the transgenics incubated with or without 20 mM or 100 mM sucrose. Methylene blue-stained *rRNA* (**C**) and CBB-stained large subunit (LSU) of RuBisCO (**D**) were used for loading controls. GUS protein was detected with anti-GUS antibody (abcam, UK).
(TIF)

Figure S2 **SC-uORF is highly conserved in higher plants.** Almost all members of plants harbor 3–5 members of the group S bZIP genes carrying the highly conserved SC-uORFs per organism. Redundant and non-redundant databases were screened by using tblastn and later analyzed manually. Sequence alignment was carried out by multiple sequence alignment software ClustalW in default parameters and then edited with Jalview editor (http://www.jalview.org/training.html). Abbreviation: *Ac - Allium cepa, At - Arabidopsis thaliana, Am - Artemisia amuna, Ah - Arachis hypogaea, Aa - Artemisia amuna, Ao - Asparagus officinalis, Bv - Beta vulgaris, Bn - Brassica napus, Bo - Brassica oleracea, Br - Brassica rapa, Cc - Citrus clementina, Cp - Carica papaya, Ci - Cichorium intybus, Cic - Citrus clementina, Cr - Citrus reticulate, Cs - Citrus sinensis, Coc - Coffea canephora, Et - Eragrostis tef, Ee - Euphorbia escula, Fa - Festuca arundinacea, Fv - Fragaria vesca, Gm - Glycine max, Ga - Gossypium arboretum, Gh - Gossypium hirsutum, Gr - Gossypium raimondi, Ha - Helianthus annuus, Ht - Helianthus tuberosus, Hv - Hordeum vulgare, In - Ipomoea nil, Ls - Lactuca sativa, Lj - Lotus japonicus, Md - Malus x domestica, Me - Manihot esculenta, Mt - Medicago truncatula, Mc - Mesembryanthemum crystallinum, Nt - Nicotiana tabacum, Os - Oryza sativa, Pv - Panicum virgatum, Pc - Phaseolus coccineus, Phv - Phaseolus vulgaris, Pt - Poncirus trifoliate, Pn - Populus nigra, Pot - Populus tremula x Populus tremuloides, Potri - Populus trichocarpa, Pa- Prunus armeniaca, Pd - Prunus dulcis, Pp - Prunus persica, Rr - Raphanus raphanistrum, Rs - Raphanus sativus, Rc - Ricinus communis, So - Saccharum officinarum, Sc - Secale cereale, Sl - Solanum lycopersicum, St - Solanum tuberosum, Sb - Sorghum bicolor, Tc - Theobroma cacao, Tp - Triphysaria pusilla, Tv - Triphysaria versicolor, Ta - Triticum aestivum, Tt - Triticum turgidum, Vu - Vigna unduiculata, Vv - Vitis vinifera, Zm - Zea mays*.
(TIF)

Figure S3 **Confirmation of SIRT in tobacco** ***tbz17*** **cDNA. A**, Schematic drawing of the cloned fragment in binary vector construct. The *tbz17* cDNA fragment (+1 to +358) was placed under the control of CaMV-35S promoter and translationally fused to *GUS* gene. **B**, Histochemical staining of the *tbz17* 5′-leader GUS transgenic plants. Among the tobacco transgenic plants, two independent lines (#1–1 and #3–1) were used for the assay. Two-week-old tobacco seedlings were incubated with or without sucrose, glucose and fructose for 2 days, then rinsed with distilled water twice, and subjected to histochemical staining according to the procedure described by Jefferson [4]. **C** and **D**, relative *GUS* transcript levels (**C**) and GUS protein levels (**D**) in the transgenics incubated with or without sugars. PCR amplification of *EF-1α* cDNA (**C**) and CBB staining of large subunit (LSU) of RuBisCO (**D**), respectively, were used for loading controls. GUS protein was detected with anti-GUS antibody (abcam, UK).
(TIF)

Figure S4 **Generation of transgenic** ***Arabidopsis*** **plants overexpressing** ***AtbZIP53*** **.** **A**, *AtbZIP53* cDNA and construction of the binary vector, pBI-AtbZIP53. **B**, Growth phenotype of 6-day-old representative *Arabidopsis* seedlings of wild-type (WT) and transgenic plants overexpressing *AtbZIP53* (*AtbZIP53-ox*). **C**, RNA blot hybridization of *AtbZIP53* in WT and *AtbZIP53-ox* plants. *AtbZIP53* endogenous-transcripts and the transgene-derived transcripts were indicated by *e* and *t*, respectively. The open reading frame (ORF) of *AtbZIP53* was used as a hybridization probe. **D**, Sugar contents in leaves of wild-type plants (WT) and 3 transgenic lines. The error bar represents the SD. The differences in sucrose contents between WT, pBI and *AtbZIP53-ox* lines were highly significant as calculated by Students *t*-test (*P<0.05; **P<0.01). **E**, RT-PCR analysis on 4 kinds of sucrose phosphate synthase (*SPS*) genes (At5g20280, At5g11110, At1g04920, At4g10120) and *FBPase* (At1g43670) genes. *Tubulin* cDNA was amplified as a control.
(TIF)

Figure S5 **Phylogenetic analysis of** ***N. tabacum*** **and** ***Arabidopsis*** **SPS protein sequences.** An unrooted neighbor-joining tree was constructed. Accession numbers of *N. tabacum* SPS cDNAs and AGI codes for *Arabidopsis* sequences are as follows: NtSPSA (AF194022), NtSPSB (DQ213015), NtSPSC (DQ213014), AtSPS1F (At5g20280), AtSPS2F (At5g11110), AtSPS3F (At1g04920), AtSPS4F (At4g10120).
(TIF)

Figure S6 **AtbZIP53 transactivates** ***ASN1*** **gene but not 4 kinds of** ***SPS*** **genes.** **A**, Schematic drawing of two effectors and 5 reporter plasmids. Those constructs were generated using the primers shown in Table S2. Horizontal short bars inside the 700-bp promoter fragments indicate the transcriptional start sites. **B**, Transactivation activity assays of AtbZIP53. Effector, reporter and Renilla LUC reference plasmids were co-bombarded into Arabidopsis mature rosette leaves by a particle delivery system (PDS-1000 He, Bio-Rad, Hercules, CA). After 18 h of incubation at 23^o^C under darkness, relative luciferase (LUC/RLUC) activities were determined. The values obtained from three independent experiments in duplicate assays were calculated with the means+SD. **P<0.01.
(TIF)

Table S1 **The primers used in this study.**
(RTF)

Table S2 **The primers used for transactivation assay.**
(DOC)

Methods S1 **Supplemental Methods.** Generation of transgenic Arabidopsis plants overexpressing *AtbZIP53.*
(RTF)
