# Supplementary figures and images for: Deregulation of Sucrose-Controlled Translation of a bZIP-Type Transcription Factor Results in Sucrose Accumulation in Leaves
Source: PLoS One. 2012 Mar 22;7(3):e33111. doi: 10.1371/journal.pone.0033111 (PMC3310857; doi:10.1371/journal.pone.0033111)

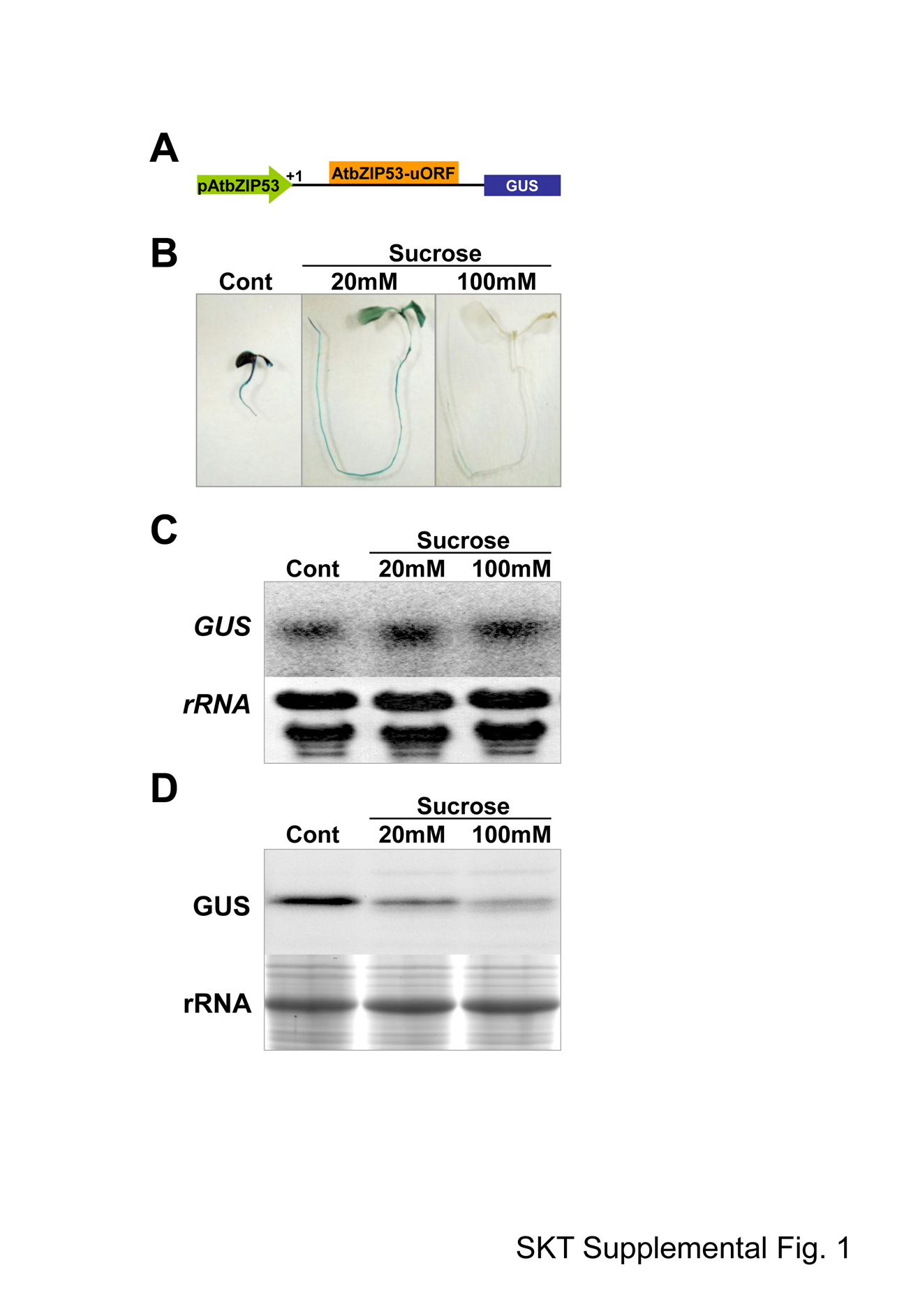

Supplement: Figure S1 — Confirmation of SIRT in Arabidopsis AtbZIP53 cDNA. A, Schematic drawing of the cloned fragment in binary vector construct for transformation. AtbZIP53 genome DNA fragment, spanning from -919 to +552, was inserted into PstI and BamHI-digested pBI101 vector (Clontech), yielding pAtZIP53G. The recombinant was a GUS-translational fusion construct. B, Histochemical staining of the AtbZIP53 promoter-GUS transgenic Arabidopsis seedlings. Among the transgenics, two independent lines were used for assay. 5-day-old Arabidopsis seedlings were incubated with or without 20 mM or 100 mM sucrose for 2 days, then rinsed with distilled water twice, and subjected to histochemical staining according to the procedure described by Jefferson (1987). C and D, GUS transcript levels (C) and GUS protein levels (D) in the transgenics incubated with or without 20 mM or 100 mM sucrose. Methylene blue-stained rRNA (C) and CBB-stained large subunit (LSU) of RuBisCO (D) were used for loading controls. GUS protein was detected with anti-GUS antibody (abcam, UK). (TIF) [file pone.0033111.s001.tif]

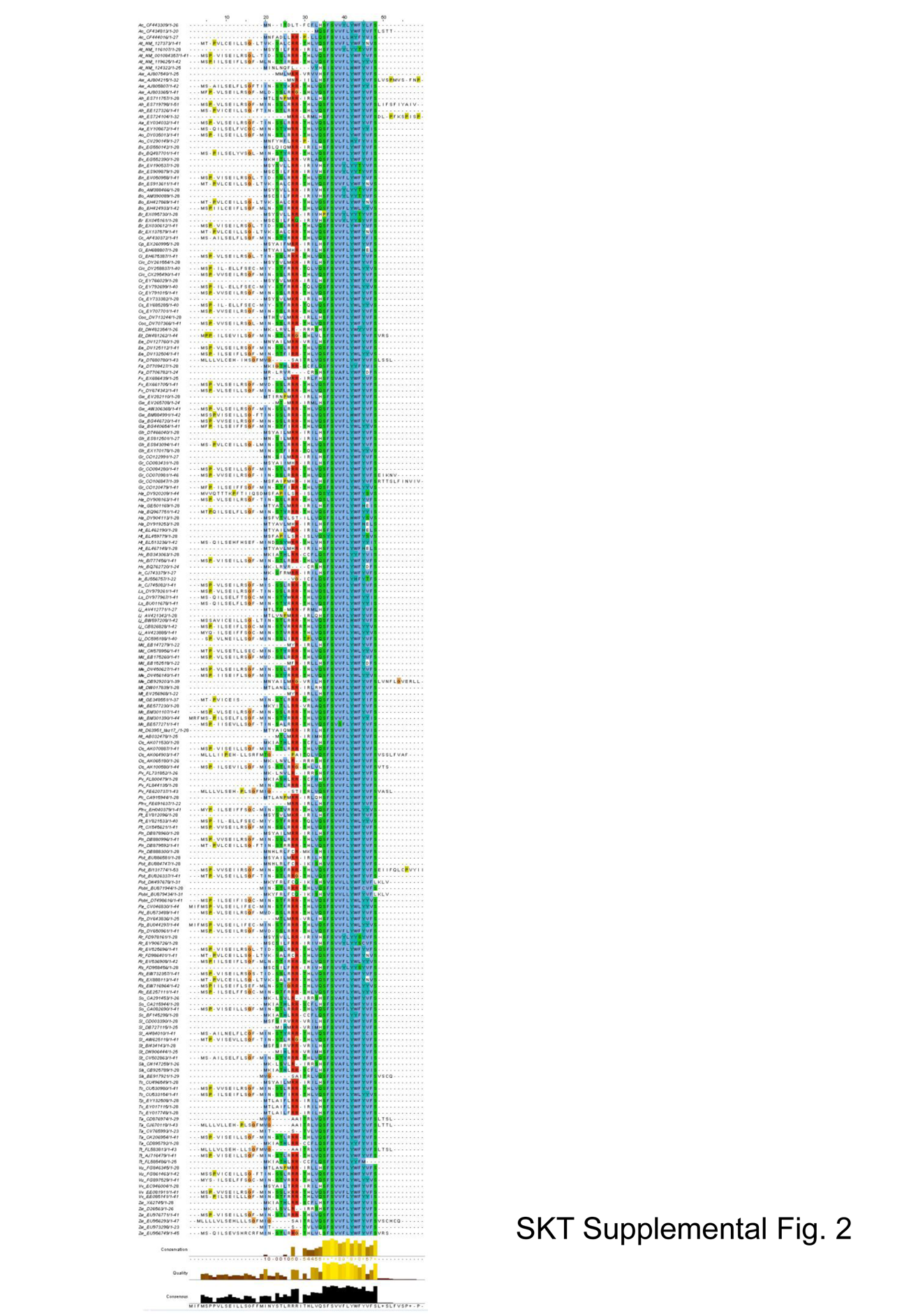

Supplement: Figure S2 — SC-uORF is highly conserved in higher plants. Almost all members of plants harbor 3–5 members of the group S bZIP genes carrying the highly conserved SC-uORFs per organism. Redundant and non-redundant databases were screened by using tblastn and later analyzed manually. Sequence alignment was carried out by multiple sequence alignment software ClustalW in default parameters and then edited with Jalview editor (http://www.jalview.org/training.html). Abbreviation: Ac - Allium cepa, At - Arabidopsis thaliana, Am - Artemisia amuna, Ah - Arachis hypogaea, Aa - Artemisia amuna, Ao - Asparagus officinalis, Bv - Beta vulgaris, Bn - Brassica napus, Bo - Brassica oleracea, Br - Brassica rapa, Cc - Citrus clementina, Cp - Carica papaya, Ci - Cichorium intybus, Cic - Citrus clementina, Cr - Citrus reticulate, Cs - Citrus sinensis, Coc - Coffea canephora, Et - Eragrostis tef, Ee - Euphorbia escula, Fa - Festuca arundinacea, Fv - Fragaria vesca, Gm - Glycine max, Ga - Gossypium arboretum, Gh - Gossypium hirsutum, Gr - Gossypium raimondi, Ha - Helianthus annuus, Ht - Helianthus tuberosus, Hv - Hordeum vulgare, In - Ipomoea nil, Ls - Lactuca sativa, Lj - Lotus japonicus, Md - Malus x domestica, Me - Manihot esculenta, Mt - Medicago truncatula, Mc - Mesembryanthemum crystallinum, Nt - Nicotiana tabacum, Os - Oryza sativa, Pv - Panicum virgatum, Pc - Phaseolus coccineus, Phv - Phaseolus vulgaris, Pt - Poncirus trifoliate, Pn - Populus nigra, Pot - Populus tremula x Populus tremuloides, Potri - Populus trichocarpa, Pa- Prunus armeniaca, Pd - Prunus dulcis, Pp - Prunus persica, Rr - Raphanus raphanistrum, Rs - Raphanus sativus, Rc - Ricinus communis, So - Saccharum officinarum, Sc - Secale cereale, Sl - Solanum lycopersicum, St - Solanum tuberosum, Sb - Sorghum bicolor, Tc - Theobroma cacao, Tp - Triphysaria pusilla, Tv - Triphysaria versicolor, Ta - Triticum aestivum, Tt - Triticum turgidum, Vu - Vigna unduiculata, Vv - Vitis vinifera, Zm - Zea mays. (TIF) [file pone.0033111.s002.tif]

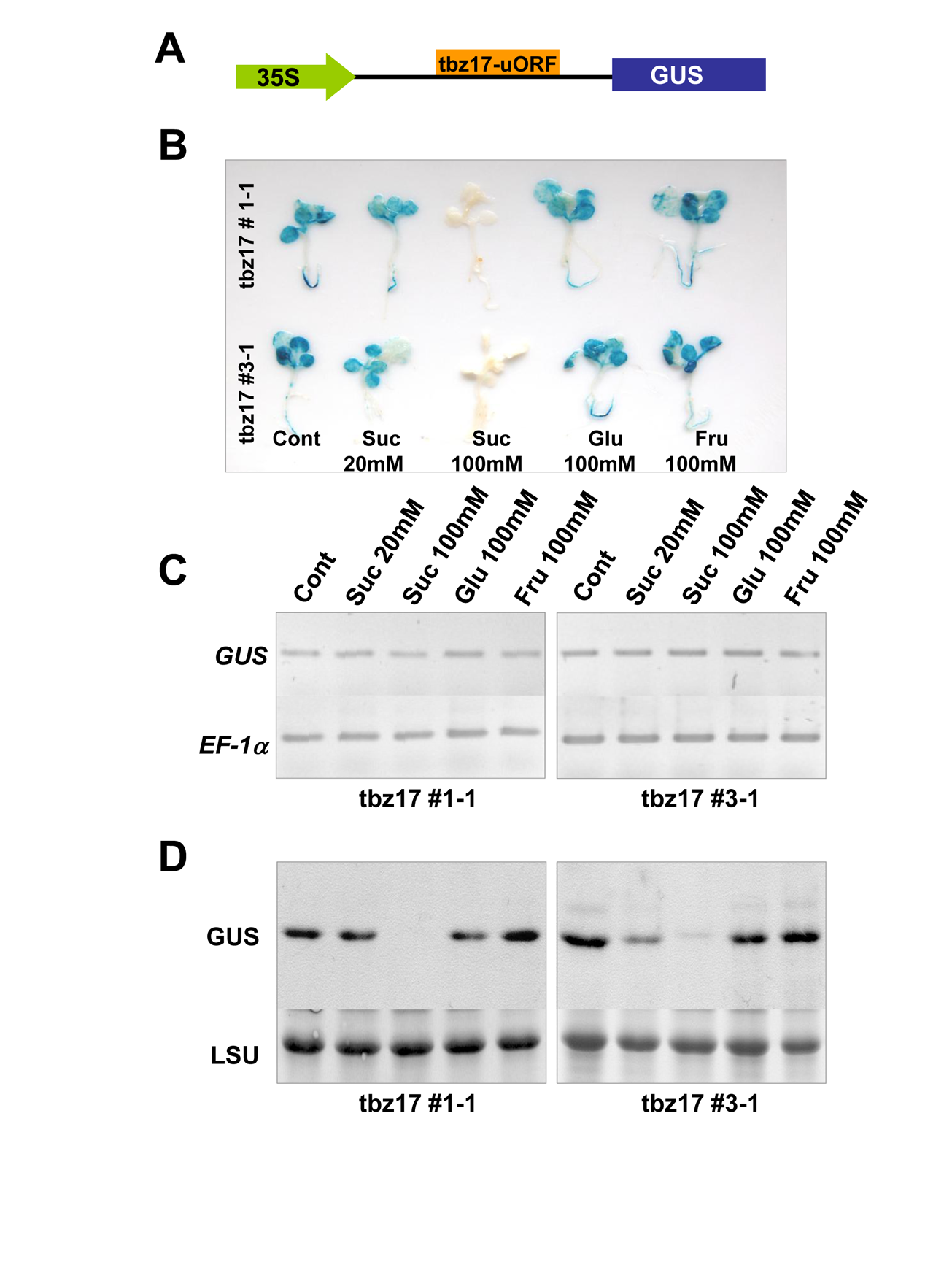

Supplement: Figure S3 — Confirmation of SIRT in tobacco tbz17 cDNA. A, Schematic drawing of the cloned fragment in binary vector construct. The tbz17 cDNA fragment (+1 to +358) was placed under the control of CaMV-35S promoter and translationally fused to GUS gene. B, Histochemical staining of the tbz17 5′-leader GUS transgenic plants. Among the tobacco transgenic plants, two independent lines (#1–1 and #3–1) were used for the assay. Two-week-old tobacco seedlings were incubated with or without sucrose, glucose and fructose for 2 days, then rinsed with distilled water twice, and subjected to histochemical staining according to the procedure described by Jefferson [4]. C and D, relative GUS transcript levels (C) and GUS protein levels (D) in the transgenics incubated with or without sugars. PCR amplification of EF-1α cDNA (C) and CBB staining of large subunit (LSU) of RuBisCO (D), respectively, were used for loading controls. GUS protein was detected with anti-GUS antibody (abcam, UK). (TIF) [file pone.0033111.s003.tif]

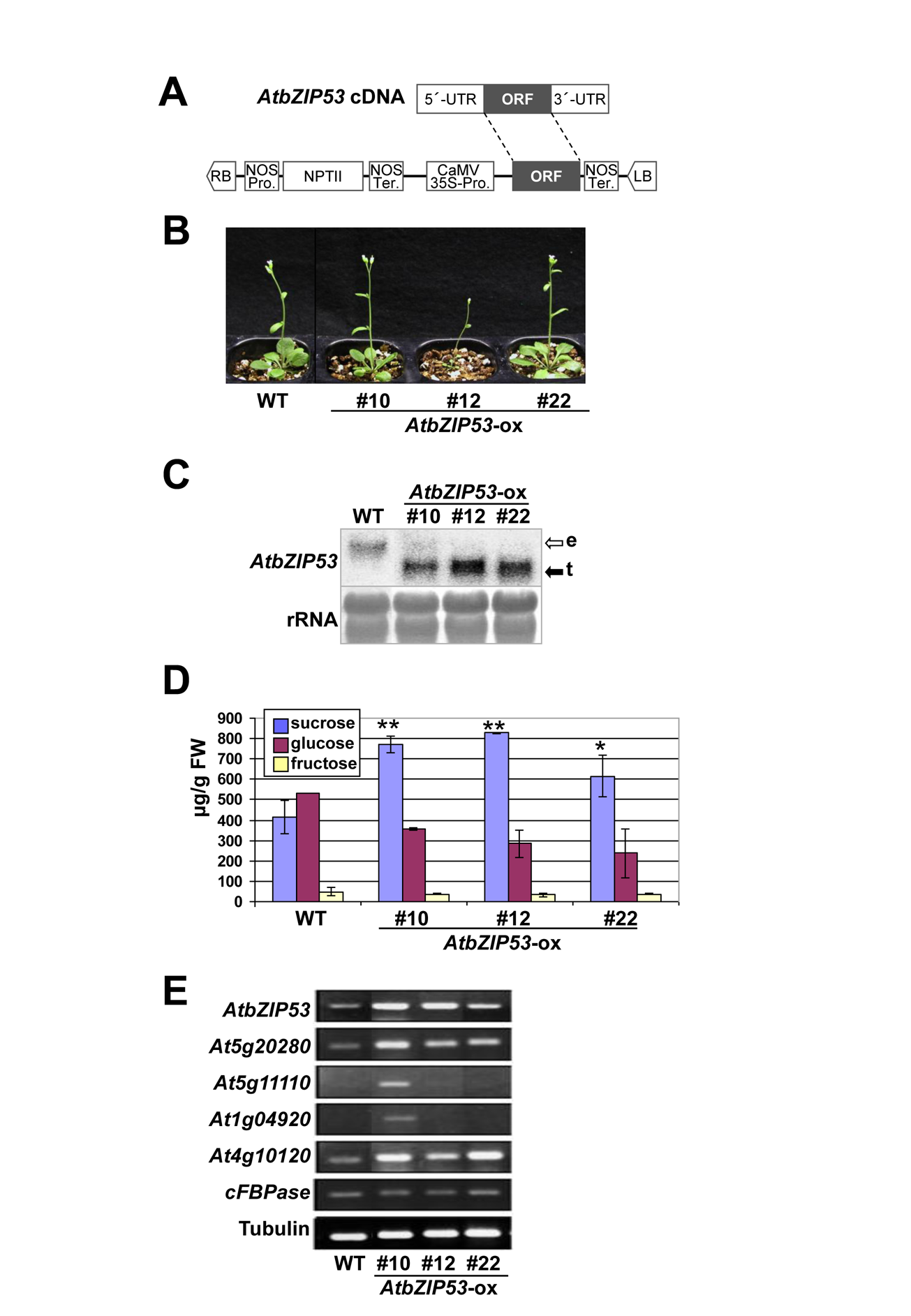

Supplement: Figure S4 — Generation of transgenic Arabidopsis plants overexpressing AtbZIP53 . A, AtbZIP53 cDNA and construction of the binary vector, pBI-AtbZIP53. B, Growth phenotype of 6-day-old representative Arabidopsis seedlings of wild-type (WT) and transgenic plants overexpressing AtbZIP53 (AtbZIP53-ox). C, RNA blot hybridization of AtbZIP53 in WT and AtbZIP53-ox plants. AtbZIP53 endogenous-transcripts and the transgene-derived transcripts were indicated by e and t, respectively. The open reading frame (ORF) of AtbZIP53 was used as a hybridization probe. D, Sugar contents in leaves of wild-type plants (WT) and 3 transgenic lines. The error bar represents the SD. The differences in sucrose contents between WT, pBI and AtbZIP53-ox lines were highly significant as calculated by Students t-test (*P<0.05; **P<0.01). E, RT-PCR analysis on 4 kinds of sucrose phosphate synthase (SPS) genes (At5g20280, At5g11110, At1g04920, At4g10120) and FBPase (At1g43670) genes. Tubulin cDNA was amplified as a control. (TIF) [file pone.0033111.s004.tif]

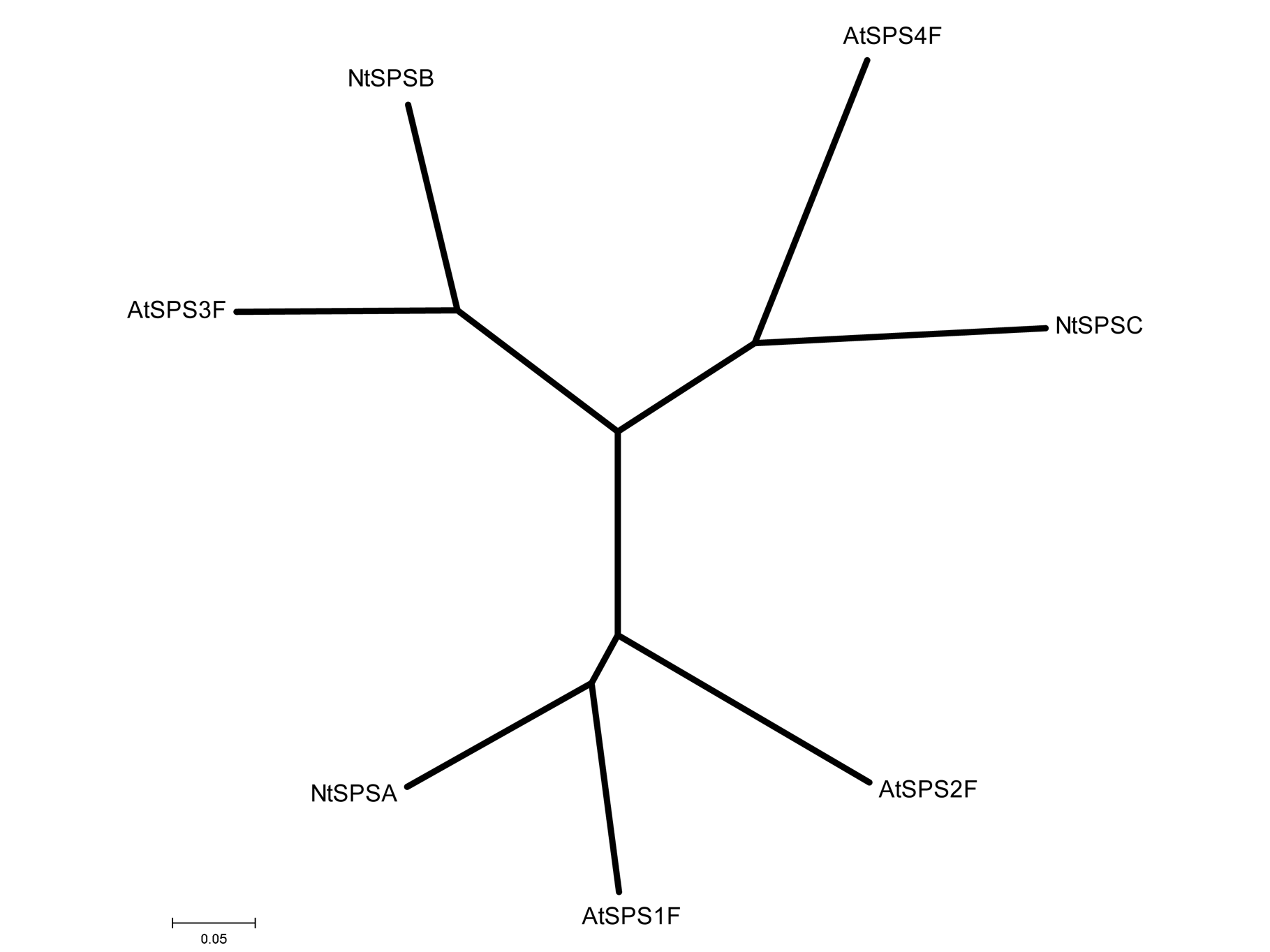

Supplement: Figure S5 — Phylogenetic analysis of N. tabacum and Arabidopsis SPS protein sequences. An unrooted neighbor-joining tree was constructed. Accession numbers of N. tabacum SPS cDNAs and AGI codes for Arabidopsis sequences are as follows: NtSPSA (AF194022), NtSPSB (DQ213015), NtSPSC (DQ213014), AtSPS1F (At5g20280), AtSPS2F (At5g11110), AtSPS3F (At1g04920), AtSPS4F (At4g10120). (TIF) [file pone.0033111.s005.tif]

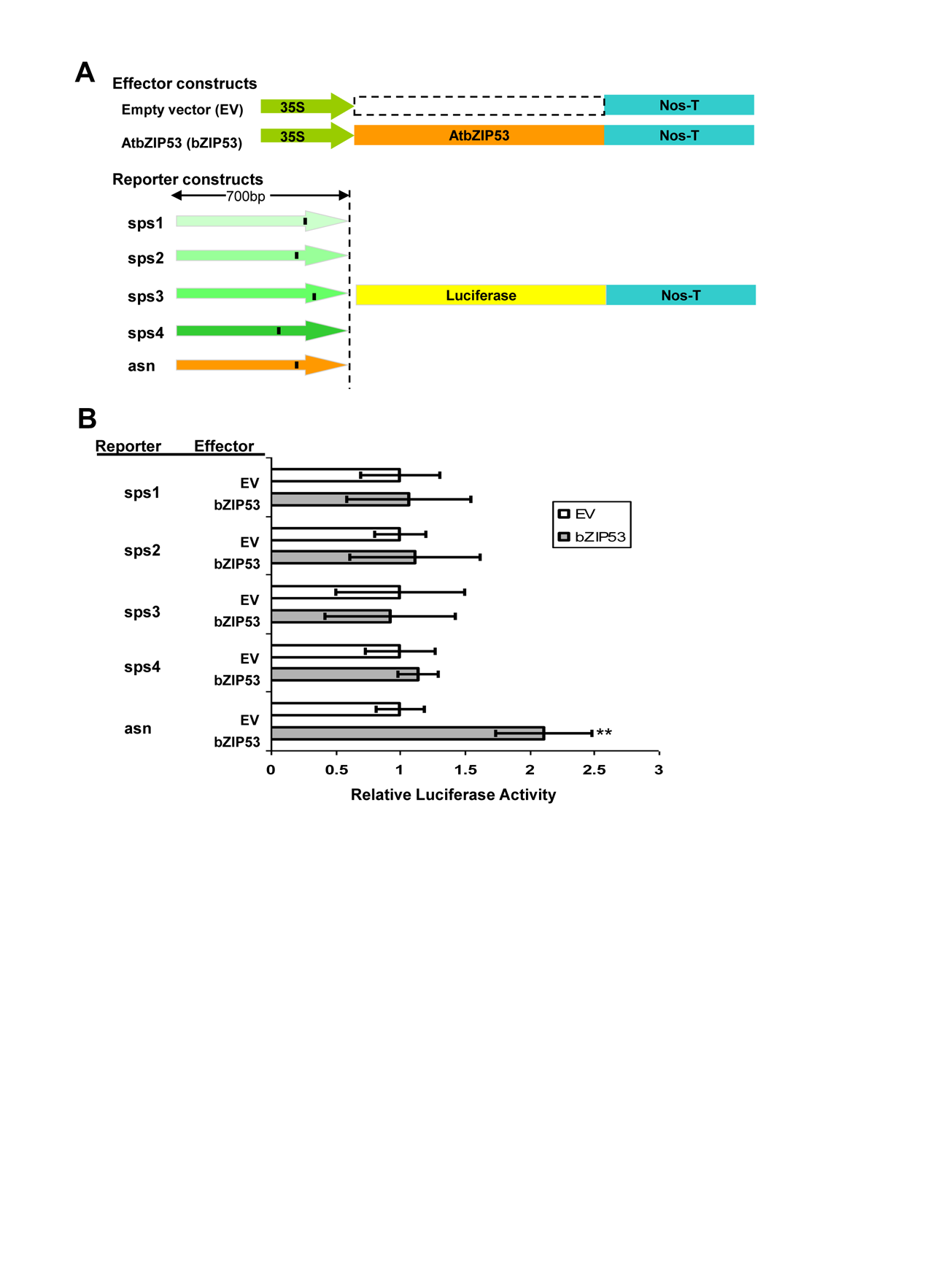

Supplement: Figure S6 — AtbZIP53 transactivates ASN1 gene but not 4 kinds of SPS genes. A, Schematic drawing of two effectors and 5 reporter plasmids. Those constructs were generated using the primers shown in Table S2. Horizontal short bars inside the 700-bp promoter fragments indicate the transcriptional start sites. B, Transactivation activity assays of AtbZIP53. Effector, reporter and Renilla LUC reference plasmids were co-bombarded into Arabidopsis mature rosette leaves by a particle delivery system (PDS-1000 He, Bio-Rad, Hercules, CA). After 18 h of incubation at 23oC under darkness, relative luciferase (LUC/RLUC) activities were determined. The values obtained from three independent experiments in duplicate assays were calculated with the means+SD. **P<0.01. (TIF) [file pone.0033111.s006.tif]
