# Supplementary material for: Deregulation of Sucrose-Controlled Translation of a bZIP-Type Transcription Factor Results in Sucrose Accumulation in Leaves
Source: PLoS One. 2012 Mar 22;7(3):e33111. doi: 10.1371/journal.pone.0033111 (PMC3310857; doi:10.1371/journal.pone.0033111)
Supplement: Table S1 — The primers used in this study. (RTF) [file pone.0033111.s007.rtf]

Supporting Information
Supplemental Table 1. The primers used in this study
Primer Name	       Primer Sequence (5 '- 3')	  Accession Number	
							
(N. tabacum genes involved in sucrose and amino acid metabolism)	
Ntsusy2_F:	CGCATAAAGGAGCAAGGACT	AB055497	
Ntsusy2_R:	TTTGCCACATCCTCAGTGAA			
Ntspsa_F:	CGGAAGTTCGAAAGAGCAAG	AF194022	
Ntspsa_R:	GAAAGATGAGGCCAGGATGA		
Ntspsc_F:	TTGTACGGTCAGGTGGCATA		DQ213014	
Ntspsc_R:	TGGCTTTTTGATCATGTGGA			
Ntspp1_F:	AAATGATAAGTGGGATCGGAAA	AY729655	
Ntspp1_R:	GGCAAAATGTCCAAATCCAT			
Ntspp2_F:	TGGTGTTGAGAAATCCCTCC		AY729656	
Ntspp2_R:	GCCATGTCTGATGTACGTGG			
Ntfbpase_F:	TAGGGCTTGCTGGTGAGACT		DV161945	
Ntfbpase_R:	TTGGAAGATCCATCCAGAGG			
Ntprodh1_F:	GAAAATAAGCTATGTGCGTAGCC	AY639145.1	
Ntprodh2_F:	GTAGTATCTTTTTCCCTATGCG	AY639146.1	
Ntprodh_R:	CAACATTTCGGCTCCACTTTG			
Ntasn_F:	AACATCTGCATGGGGCTTAG		AY061820.1	
Ntasn_R:	TGCTTTGGGAGATAGGGATG			
Nttbz17_F:	TGCAGACCAGCAGTATGCAAAGTT	D63951.1	
Nttbz17_R:	TCCTCTCGCGGCAGATACTGT		
Ntpolyubi_F:	ATTCGGCACGAGGAAAGAC		AF154647	
Ntpolyubi_R:	TGGATGTTGTAATCGGCTAGG		
NtL25_F:	CTAAGGTTGCCAAGGCTGTC		L18908	
NtL25_R:	TTGCAGACTCTGTGGTGAGG			
(N. benthamiana genes involved in sucrose and amino acid metabolism)	
Nbsusy2_F:	CGCATAAAGGAGCAAGGACT	G0602500	
Nbsusy2_R:	TTTGCCACATCCTCAGTGAA			
Nbspsa_F:	GCTGTTTTGTCCTGGTCTAAGG	HQ844225	
Nbspsa_R:	GGAACCCTCTGCTCTTTCTTTC		
Nbspsc_F:	TGAGTCTGACCTCCACAAGGCTTGG	G0603653	
Nbspsc_R:	CGGGCAAGATGCCAAATGCGC		
Nbspp_F:	GCACGTCTCATGATAGTCTCAGATC	G0603390	
Nbspp_R:	GTAGGTGATCTCCCAGTTGAGAAC		
Nbfbpase_F:	TCCATACATGCAGTATCCAGCAGCTAAC	G0612973	
Nbfbpase_R:	TGTGTAGTTTTTGATCCTCTGGATG		
Nbprodh_F:	AACCTCGGATTTCATGTCCGCC	G0603506	
Nbprodh_R:	CAACTTTGAGGTAGGAACGCCG		
Nbasn_F:	AACTTCTGCATGGGGCTTAG		CK281644	
Nbasn_R:	TGCTTTGGGAGATAGGGATG			
Nbtbz17_F:	GCTCACTCAATTTTGGGCT		FN908499	
Nbtbz17_R:	AAGAGGCATCGATAGGTTGA			


Supplemental Table 1 (Continued)

Primer Name	        Primer Sequence (5 '- 3')	Accession Number	
NbL25_F:	CTGACCCCAAAGCACAGGCAGC	ES888796	
NbL25_R:	CCTTCCAGGTGCACTGATACGAGGG		
(Arabidopsis thaliana genes' primers)	
AtbZIP53_F:	GCATGGGGTCGTTGCAAATGC	At3g62420	
AtbZIP53_R:	CGTCAGCAATCAAACATATC			
Atsps1F_F:	CGTGATGCCCTGAAGAAATTAGAGCTT	At5g20280	
Atsps1f_R:	GGAATGGTTTTATTTATATGAATGTTGTTC	
Atsps2F_F:	GGAATTCACAAAACAGTGATACTTAAAGGC	At5g11110	
Atsps2F_R:	GCATAAGATTTCATATGTCATCAATCCTAT	
Atsps3F_F:	CGTCACATTGGGTTCTGATGCTCTTC	At1g04920	
Atsps3F_R:	GCCTCGACTTCACTATTTGAAAATTTTATT	
Atsps4F_F:	CCATCATTCTAAAAGGTGTAGTGGGAT	At4g10120	
Atsps4F_R:	GCGTTTTATTGAATCTATACTAAGCAAG		
Atcfbpase_F"	GGAAAATTGCGTGTCTTGTATGAAGTTTC	At1g43670	
Atcfbpase_R"	CCTTTTGTAAATGAGTTATATTGTTCTTCT		
Attubulin_F:	CGTGGATCACAGCAATACAGAGCC	At5g23860	
Attubulin_R:	CCTCCTGCACTTCCACTTCGTCTTC		
Attubulin_qF	GGTGAAGGAATGGACGAGATG	At5g23860	
Attubulin_qR	CTGCACTTCCACTTCGTCTTC		
Primers used for Luc and RLuc transcripts		
FLuc_F:	AGCGACCAACGCCTTGATT			
FLuc_R:	TCCCAGTAAGCTATGTCTCCAGAA		
RLuc_F:	ATGGGATGAATGGCCTGATA			
RLuc_R:	GCTGCAAATTCTTCTGGTTCT			
Primers used for Gus transcript			
gus_F:	TATACCGAAAGGTTGGGCAG			
gus_R:	TCGTTGTTCACACAAACGGT			
