# Supplementary material for: Deregulation of Sucrose-Controlled Translation of a bZIP-Type Transcription Factor Results in Sucrose Accumulation in Leaves
Source: PLoS One. 2012 Mar 22;7(3):e33111. doi: 10.1371/journal.pone.0033111 (PMC3310857; doi:10.1371/journal.pone.0033111)
Supplement: Table S2 — The primers used for transactivation assay. (DOC) [file pone.0033111.s008.doc]

**Supplemental Table 2.** The primers used for transactivation assay

| **Primer Name Primer Sequence (5'- 3')** | | | **Accession number** | | | |  | |
| --- | --- | --- | --- | --- | --- | --- | --- | --- |
| *bZIP53F* | acctctagaTCGTTCTCGATCTCAGTTTTCG |  | | At3g62420 |  |  | |  |
| *bZIP53R* | catgagctcACAAATTGAAATTGCATAAA | | | | | | |  |
| *AtasnF* | catctgcagTAACTTTACTTCTCATTTTT At3g47340 | | | | | | |  |
| *AtasnR* | acctctagaGTTTTTTTTTTGAAGAAAGTG | | | | | | |  |
| *Atsps1F* | catctgcagATGATGGGAAAAAGAGACAT At5g20280 | | | | | | |  |
| *Atsps1R* | acctctagaCTGGTGGGACGATCAAAGAT | | | | | | |  |
| *Atsps2F* | catctgcagTTGATAATATCTGATTTTGA At5g11110 | | | | | | |  |
| *Atsps2R* | accggatccTGGTGTTCGCTCTCCCGAAC | | | | | | |  |
| *Atsps3F* | catctgcagCAACATACGTGTAGTAATAG At1g04920 | | | | | | |  |
| *Atsps3R* | acctctagaTCTGAGTTTGTCCTCTGTTT | | | | | | |  |
| *Atsps4F* | catctgcagATATTACACTCATCTTTCTC At4g10120 | | | | | | |  |
| *Atsps4R* | acctctagaTCTCTGATTGTTGCACTGA | | | | | | |  |
|  |  | | | | | | |  |

The small letter sequences were added for cloning purpose.
