# Supplementary material for: Deregulation of Sucrose-Controlled Translation of a bZIP-Type Transcription Factor Results in Sucrose Accumulation in Leaves
Source: PLoS One. 2012 Mar 22;7(3):e33111. doi: 10.1371/journal.pone.0033111 (PMC3310857; doi:10.1371/journal.pone.0033111)
Supplement: Methods S1 — Supplemental Methods. Generation of transgenic Arabidopsis plants overexpressing AtbZIP53. (RTF) [file pone.0033111.s009.rtf]

Supplemental Methods
Generation of transgenic Arabidopsis plants overexpressing AtbZIP53 
The coding region of AtbZIP53 was amplified by PCR using a pair of gene-specific primers (forward, 5'-GCGGATCCATGGGGTCGTTGCAAATGC-3' and reverse, 5'-CGGAGCTCTCAGCAATCAAACATATC-3') and the AtbZIP53 cDNA as a template. The fragment digested with BamHI and SacI was inserted into the respective sites of the pBI121 vector (Clontech), resulting in p35S::AtbZIP53. This plasmid was introduced into Agrobacterium tumefaciens EHA105 [1] cells by electroporation [2]. A. thaliana ecotype Col-0 plants were transformed by the floral dip-method [3] using the A. tumefaciens culture. Transformants were selected on MS agar medium containing 50 ìg/ml kanamycin. Four lines of transgenic plants having a single copy of the transgene (judged by a segregation ratio of 3:1, kanamycin-resistant to -sensitive seeds), lines 10, 12 and 22, were selected for further analysis.

1. Hood EE, Gelvin SB, Melchers LS, Hoekema A (1993) New Agrobaterium helper plasmids for gene transfer to plants. Transgen Res 2:208–218.
2. Shen WJ, Forde BG (1989) Efficient transformation of Agrobaterium spp. by high voltage electroporation. Nucleic Acids Res 17:83–85.
3. Clough SJ, Bent AF (1998) Floral dip: a simplified method for Agrobacterium-mediated transformation of Arabidopsis thaliana. Plant J 16:753–743.
4. Jefferson RA (1987) Assaying chimeric genes in plants: the GUS gene fusion system. Plant Mol Biol Rep 5:387–405
